# Editorial

**Published:** 1871-12

**Authors:** 


### THE CLOSE OF THE YEAR.

Another turn of the great wheel of time, and another year has been numbered with the things of the past. It is well, at the close of the year, to take a retrospect—to seriously contemplate whether, as individuals, we have added anything to the interests of science, or have been the instruments for good in the hands of a kind Providence, to the weary and suffering of our unfortunate race. The record has been written, either for or against us, either on the bright pages of the celestial volume, to our honor and immortality at the right hand of the Father of light, or sounded by fiend to fiend along the dark corridors of that habitation where dwelleth the "devil and his angels!" Not all of earth, or of heaven, can undo one single deed of the past. Not one tracing of the burning pen of time, past and gone, can ever be eraced. Its imprintings of good or evil, of joy or woe, no power can change, no tear obliterate. With fear and trembling then, let us turn back, leaf by leaf. the written record of our lives, numbered by the hours and days of the past. Ah! we fear there will be found few items in the great account, to our honor or to our praise, judged by the standard of a holy and pure morality. Still, let us turn the pages and read the lines teeming with guilt, it may be, or of time misspent in the employment of things impure, and of omissions of duty, the performance of which would have been for our praise, and the source of happiness to others.

But as medical journalists, we have our volume of acts to turn over, and as we go back, laaf by leaf, we find no regrets for our efforts to maintain those great, heaven-born principles which underlie all human society—the purest, soundest morality or ethics, upon which alone is founded medical law. Not a sigh, not one emotion of sorrow glooms the road of honor we marked from the beginning—save that, here and there along the pathway we have trodden, we have mentally failed to come up to the high demands and our own desires in reference to the noble principles so dear to the hearts of all good men, in all the moral grandeur and power of imperishable truth. So far as The Companion is concerned these principles shall ever be the central cluster of all its revolving hopes; from which if it should willfully swerve but for a moment, it ought to meet the frowns and the deep-toned anathamas of our enlightened and honorod profession. For the future—oh! who so profound as to draw aside the veil that covers the pathway of earth!—for the future, with our hand upon our heart, and our heart lifted high to the source of all wisdom and truth, we ardently aspire to tread the narrow way marked out by such a guide, not veering to the right or left, as we pass along the high-road of duty! In such a path-way, no demands of honor shall be hushed at the bidding of policy, that truth may be put to ignoble torture and principle chained to the car of devilish expediency.

But we forbear. With this number we close the first volume of an undertaking for the noble purpose of aiding in giving help to our professional brethren in hours of uncertainty, and by this means to aid in accomplishing the grand object of all human love and science—the alleviation of suffering humanity. We feel, in our inmost heart, that our subscribers endorse the high positions it has been the pleasure and duty of THE COMPANION to mark out in these times of moral and professional degeneracy. As the clock of time shall number the coming months of the new year, we hope, should life be spared, to add increasing interest to the medical character, and an unwavering zeal in defense of the high principles of THE COMPANION.

It will speak for the good, the true and the noble men of the profession, that honor and professional morality may triumph, and every opposing obstacle to professional integrity and dignity up-rooted and removed.

